# A New Method for the Digital Assessment of the Relative Density of Bone Tissue in Dentistry Using the ImageJ Software Package

**DOI:** 10.3390/dj13080375

**Published:** 2025-08-19

**Authors:** Mariya Ebrakhim, Denis Moiseev, Valery Strelnikov, Alaa Salloum, Ekaterina Faustova, Aleksandr Ermolaev, Yulianna Enina, Ellina Velichko, Yuriy Vasil’ev

**Affiliations:** 1 Dentistry Department, Tver State Medical University, Tver 170100, Russia; maria.ibrahim.salloum@gmail.com (M.E.); strel-stom@yandex.ru (V.S.); alaa.salloum1993@gmail.com (A.S.); 2 School of Dentistry, Pirogov Russian National Research Medical University, Moscow 117513, Russia; faustova_ee@rsmu.ru (E.F.); ermolaev2009@yandex.ru (A.E.); 3 Operative Surgery and Topography Anatomy Department, Sechenov University, Moscow 119048, Russia; enina_yu_i@staff.sechenov.ru; 4 Pathophysiology Department, Sechenov University, Moscow 119048, Russia; velichko_e_v@staff.sechenov.ru

**Keywords:** ImageJ, peri-implantitis, radiograph, relative bone density

## Abstract

**Backgroud**: The aim of this study was to create an accessible, simple and reliable method for assessing the relative density of bone tissue in dentistry based on the analysis of digital panoramic radiographs. **Methods**: Measurement of average gray values on orthopantomograms was carried out using ImageJ Version 1.54i software. To estimate the relative bone density, functions for selecting regions of interest (ROI), calculating the area of selection, and statistics of the selected area were used. Statistical characteristics of samples and testing of hypotheses using statistical criteria were performed using Microsoft Excel. **Results**: we found that when manually selecting the reference and comparison areas for areas without signs of pathological changes in bone tissue, the average standard deviation was 0.058, and the coefficient of variation was 0.055 ± 0.011%, which makes the choice of the jaw angle as a reference more preferable. The average relative bone density of the assessed defective areas to the jaw angle was 0.64 ± 0.11, and the average relative bone density of the areas without pathology to the jaw angle was 1.052 ± 0.058. **Conclusions**: a research protocol was developed and justified using the ImageJ software package, which establishes a strict procedure for quantitative assessment of relative bone density based on the results of digital panoramic radiography. The proposed protocol can be used to monitor the condition of bone tissue after all types of dental treatment over time.

## 1. Introduction

In daily dental practice, there is a need to determine the degree of healing of bone tissue after various types of interventions, to assess the effectiveness of such treatment and plan its further stages. The quality of jaw bone tissue plays an important role in treatment planning in implantology. To determine the maturity of regenerated bone, the most common method currently is X-ray examination [1,2].

X-ray diagnostics are indispensable in the practice of a dentist and, along with clinical data, allow for a comprehensive assessment of the condition of the dental system and the maxillofacial area as a whole. Such diagnostics are used to identify hidden carious cavities, to investigate periapical pathology, to assess the condition of the periodontium and tissues surrounding the implant, to assess the morphology of the roots of the teeth, trauma to the teeth and associated structures, and in planning and evaluating the results of dental implants [3,4,5,6,7,8,9].

The primary X-ray examination available to a practicing physician is orthopantomography (digital or film), which allows one to obtain a detailed image of all parts of the maxillofacial region with adjacent parts of the facial skeleton. To assess bone density, various densitometry methods are used, as well as computer tomographs, which provide the ability to assess bone density according to the Hounsfield scale. The Hounsfield Units Scale quantitatively displays the ability of various objects to attenuate X-rays, and includes 4096 values from −1024 to +3071 Hounsfield units (HU). Bone tissue density according to the Hounsfield scale ranges from 250 to 3000 HU [10,11]. The use of densitometry in the work of a dentist is extremely limited due to its high cost and inaccessibility. Therefore, computed tomography remains the preferred method for assessing the relative density of bone tissue [12].

Unlike computed tomography (CT), orthopantomography (OPTG) is a fast and inexpensive imaging technology available in almost every dental office, and it is the only imaging method that provides a complete image of the jaws, teeth, temporomandibular joints, alveoli and paranasal sinuses in one image, which gives the dentist the opportunity to study and analyze all the components of the dentofacial apparatus as a whole. The results of OPTG can serve as a foundation for analysis using image processing programs.

Medical image analysis is one of the most actively developing areas in computer processing. The ability to process images is provided by such software systems as Bioscan (UK Ltd., Leicester, UK), OpenCV (Open Source Computer Vision Library, Itseez, Russia), MATLAB, version R2024b (MathWork Inc., Natick, Massachusetts, USA), and ImageJ (Wayne Rasband, National Institute of Health, USA). Among the many software packages, ImageJ is the most accessible and preferred tool because it is a freely distributed program, available for quick study and actively updated with plugins for medical research. There many advantages found in other additional packages.

A.D. Lemos et al. used the linear and spatial measurement capabilities of ImageJ to analyze digital panoramic radiographs to differentiate between functional and morphological asymmetries of the mandible in children with and without unilateral posterior crossbite [13].

Hans R. Preus et al. aimed to propose and evaluate an indirect radiological method for measuring radiological bone level and measuring bone loss using the ImageJ software package [14].

ImageJ was used to evaluate the quality of dental root canals for various irrigation protocols. Using ImageJ plugins, a multiparameter analysis of microphoto measurements was carried out with a magnification of 750×: the treatment area and the number of dentinal tubules were calculated [15].

Nur Fadhilah et al. used ImageJ to calculate the area of periapical abscess involvement [16].

In a statistical study of the modern Malaysian population, ImageJ was used to quantify secondary dentin formation [17].

In Russian studies, ImageJ was used to evaluate shrinkage and swelling parameters of dental impression material samples, while other researchers used it to analyze abnormal dental arch shapes in orthodontic treatment planning. ImageJ was used to determine the area of protein fractals and prove the direct relationship between the numerical values of this indicator and the overall level of oral hygiene [10,18].

A digital X-ray image is represented as a collection of digital pixel values (gray scale values). During digitization of the image, each pixel is assigned a digital value from 0 to 255, in which 0 corresponds to black (low radiological density), and 255 to white (complete absorption of X-rays) [18]. Digital processing of X-ray images allows the doctor to make a diagnosis relying not only on experience but also on qualitative and quantitative measures of information.

In 2017, Khojastepour L. et al. showed in their study that the average gray scale value of digitized images is proportional to the bone density value [12], which provides a rationale for using image processing software to estimate bone density.

Indeed, ImageJ allows the user to manually select areas of interest, calculate the statistical characteristics of the selected areas, and perform batch processing. For the selected area, the number of pixels, the average gray value of the selected area (optical bone tissue density), standard deviation, maximum and minimum values, and other statistical characteristics are automatically calculated.

Direct comparison of the optical density of areas of interest in different images is impossible due to objective conditions, which include different shooting conditions and image brightness. To compare changes in the optical density of areas of interest over time, it is necessary to use a relative characteristic.

Relative bone density is determined by measuring the average gray value of an area of interest and comparing it to a healthy environment or a specific reference area that is not overlapped by other anatomical structures or associated with any pathology:

$$\mathrm{Relative}\;\mathrm{bone}\;\mathrm{density}\;=\;\frac{average\;gray\;value\;of\;area}{average\;gray\;value\;of\;healthy\;bone}$$

It is not possible to establish a reference value for healthy bone density from image processing. Even in one image, the coefficient of variation (the ratio of the standard deviation of the average gray value to the mean value) takes on different values in different areas.

Measuring bone density is a process that involves manually selecting an area of interest. A group of German scientists (Geiger M. et al.) in 2016 assessed the repeatability of results using measurements from three evaluators. Statistically significant repeatability of the results of measuring bone tissue density of the areas of interest was shown, and the average coefficient of variation was 2.972 ± 2.361%. At the same time, the coefficient of variation for areas without pathological changes was higher (3.691 ± 2.626%) [18]. This is explained not only by the method of manual selection of the analysis area (the area in the vicinity of the pathologically changed one was selected), but also by the structure of the bone tissue and the method of image creation.

Therefore, to improve the accuracy of the assessment of relative bone density and to enable correct comparisons of relative density over time, knowledge of the range of changes in the relative density of healthy bone is necessary.

Based on the above, it becomes obvious that for an effective and reliable assessment of the relative density of bone tissue, the following are necessary: a reasonable choice of the reference area and knowledge of the range of changes in the relative density of healthy areas of the jaw bone, and an assessment of the sensitivity of the “relative bone density” indicator to varying degrees of bone loss.

The purpose of the study is the development of a standardized method for assessing bone density based on the analysis of digital panoramic radiographs using the ImageJ software package.

## 2. Materials and Methods

The measurement of mean gray values on orthopantomograms was performed using ImageJ Version 1.54i software (Wayne Rasband, National Institutes of Health, Bethesda, MD, USA). To assess bone density, functions for selecting regions of interest (ROI), calculating the area of selection, and statistical data of the selected area were used.

A digital X-ray image is represented as a set of digital pixel values (gray scale values). During image digitization, each pixel is assigned a digital value from 0 to 255, where 0 corresponds to black (low radiological density) and 255 corresponds to white (complete absorption of X-rays) [18]. Digital processing of X-ray images allows the doctor to rely not only on his experience, but also on the qualitative and quantitative measure of information when making a diagnosis.

**Figure 1:**
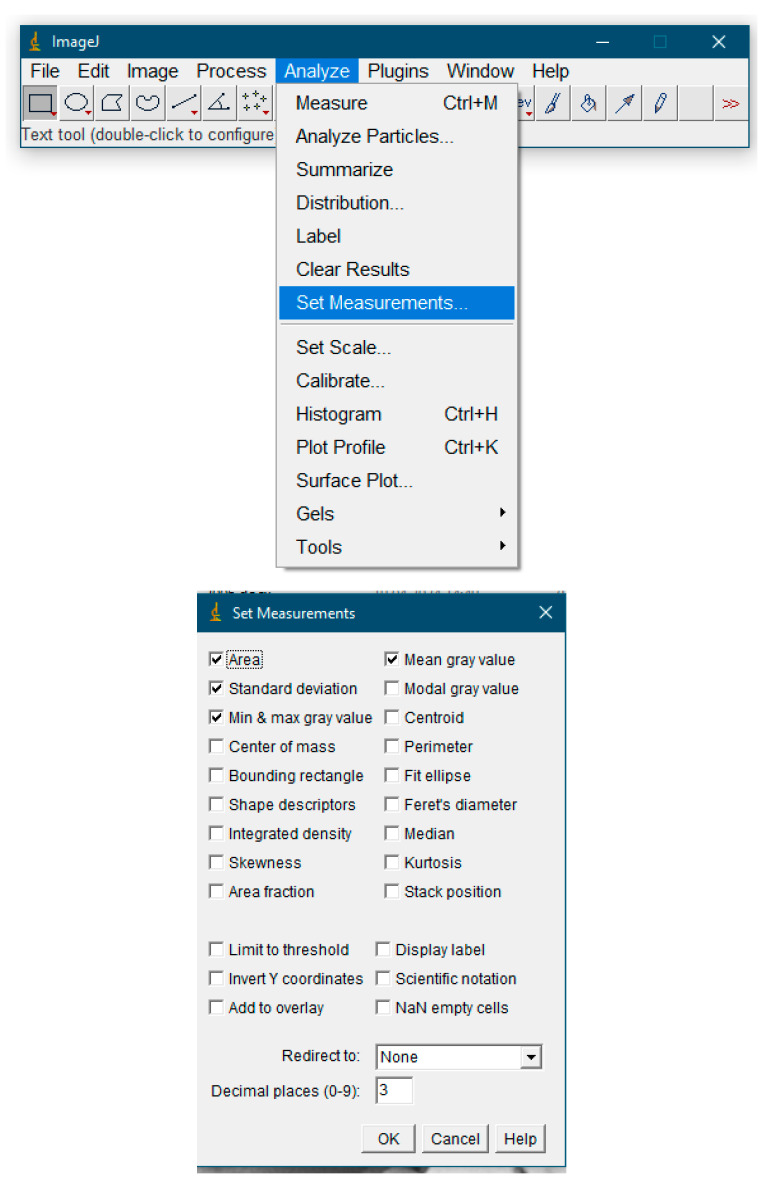
Setting measurement parameters.

Measurement procedure:

1. Set the measurement function parameters. Select the Analyze > Set Measurements menu to set the parameters that will be used in the measurements: area, average gray value, standard deviation, maximum and minimum gray value in the selected area (Figure 1).
2. Open the X-ray image file. To do this, select the File > Open menu and specify the path to the image or drag the image. If you need to improve image quality, you can use additional processing functions.

### 2.1. Image Preprocessing

All images were analyzed as is, except for the following cases:

3. When there was a visual artifact of uneven illumination (assessed by three researchers);
4. When SNR < 20 dB (calculated via Analyze → Tools → Noise Estimation);
5. When the variation coefficient was >15% in control measurements;
6. Selection of ROI (area of interest).

**Figure 2:**
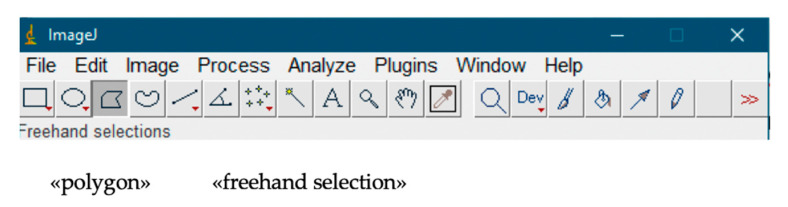
Selecting an area of the image for analysis.

The selection of an image area for analysis was performed using the «polygon» and «freehand selection» tools (Figure 2).

### 2.2. ROI Selection Standardization Protocol

A step-by-step instruction with visual markers was developed:

The jaw angle area was selected from the line connecting the lower edge of the second molar to the apex of the lower jaw angle, with an indentation of 2 mm from the cortical layer.

### 2.3. Inter-Operator Variability Assessment (ICC)

- Jaw angle: ICC = 0.982 (95% CI 0.971–0.991)
- Alveolar ridge: ICC = 0.945 (95% CI 0.912–0.968)
- Pathological areas: ICC = 0.891 (95% CI 0.832–0.931)

### 2.4. Blinded Repeated Measurement

- 30% of images were re-processed by three independent observers
- Inter-Operator Variability Coefficient
- For reference area: 1.2 ± 0.4%
- For defects: 4.7 ± 1.1%

### 2.5. Control of Operator-Dependent Variability

All measurements were duplicated by a second observer. A discrepancy of >5% in ROI area required a consensus assessment involving the project manager.

The selected area had to cover the entire radiologically visible part of the defect. The choice of the reference area had to correspond in area to the area of the defect, and the coefficient of variation had to correspond to that of the healthy area. The area located in the area of the angle of the lower jaw was chosen as a reference area. The rationale for the choice and calculation of the characteristic coefficient of variation will be given below.

When isolating an ROI according to the recommendations of Ihan Hren and Milyavets [19], it is necessary to ensure a minimum safe distance to any cortical structures (for example, the cribriform plate after tooth extraction).

If one needs to work with multiple ROIs, one must use the region of interest manager: Menu > Analyze > Tools > ROI Manager and add to Manager command. Using the Ctrl + T hotkey allows the user to add the current selection to the ROI Manager. In the area of interest manager, the user can add selections, delete, rename, save, sort selections, calculate specified parameters, etc.

**Figure 3:**
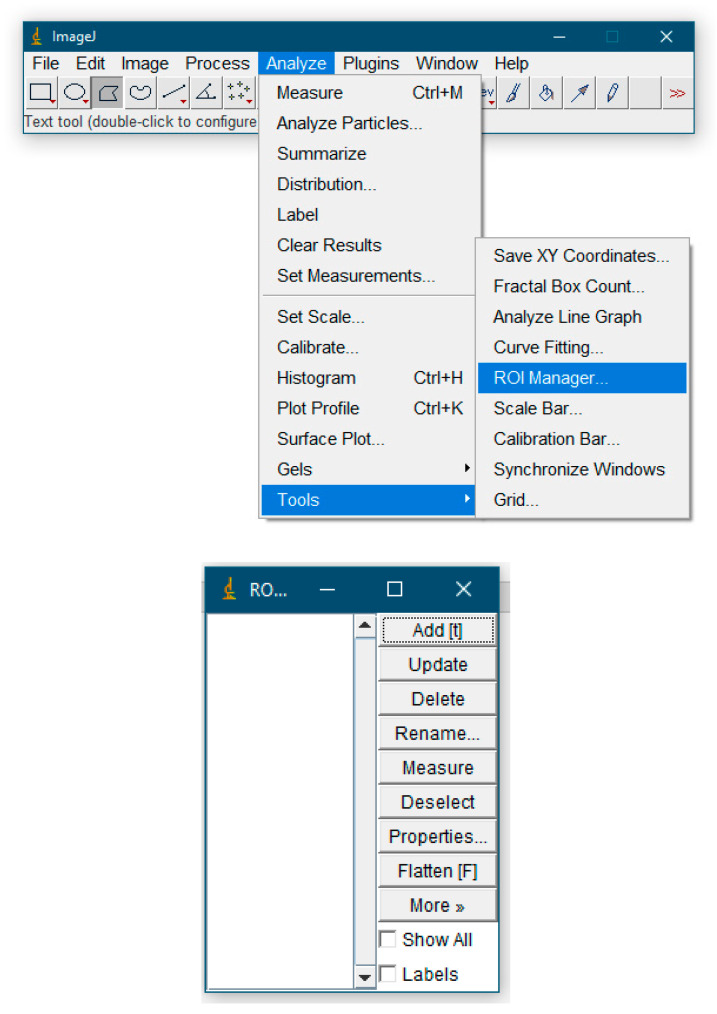
ROI Manager features.

7. Take the necessary measurements. If there is only one selection area, then the Analyze > Measure command for the previously set Set Measurements parameters in the special “Results” window will display the measurement results. If ROI Manager was used and many ROIs were selected, then to get the result one must select the Measure item (Figure 3).

To conduct this retrospective observational study with elements of pilot analysis research, images were used from the database of the clinic of Professor Strelnikov (Tver, Russia) and the Dental Clinic of the Institute of Dentistry of the Russian National Research Medical University named after N.I. Pirogov (Moscow, Russia). The main stages of the work were data collection (January 2020–December 2023), cross-sectional analysis and methodology validation.

Demographic characteristics and selection criteria: 30 subjects aged 48.2 ± 12.1 years (women 53%, men 47%). Inclusion criteria: (1) availability of panoramic radiograph before/after treatment; (2) absence of systemic bone diseases; Exclusion criteria: artifacts on the images and age < 18 years. A total of 312 images in the database were used, of which 94 met the criteria and 30 were selected using a stratified randomization method (by age/gender/pathology type).

Statistical characteristics of the samples and testing of hypotheses using statistical criteria were performed using Microsoft Excel [Microsoft Excel 2013, Microsoft Corp., Redmond, Washington, USA].

To ensure the reliability of the results, multi-level statistical analysis was used, involving: (1) primary processing in Excel with verification; (2) mixed models in R (taking into account repeated measurements); and (3) Bootstrap analysis in Python (1000 iterations), version 3.10. The normality of distribution was tested using the Shapiro–Wilk test. For pathological ROIs, the Mann–Whitney test (a non-parametric analogue of the *t*-test) was used, with correction for multiple comparisons conducted using the Benjamini–Hochberg method. Significance criterion: α = 0.05. All results remained significant. The coefficient of variation was determined at the level of CV < 10% (acceptable for medical measurements). G*Power 3.1.

In order to justify the choice of the reference area, we conducted studies of the bone tissue density of the alveolar part of the lower jaw in the area of molars and premolars without signs of pathological changes in bone tissue. The number of radiographs included in the study was 15, on which 55 areas of comparison were identified with the reference area—the angle of the mandible (Table A1).

To assess the possibility of using the “relative bone density” indicator for monitoring over time, 18 panoramic radiographs of patients with various types of changes in bone tissue were analyzed. The distribution of panoramic radiographs is shown in Table A2. A priori power analysis was performed using G*Power 3.1, for a two-sample *t*-test (comparison of healthy and pathological areas). G*Power result:

- Required sample size: 17 per group (34 measurements in total).
- Actual size in the study:
  ○ Healthy areas: 55 ROI (exceeds calculation).
  ○ Pathological areas: 18 ROI (sufficient).

Sensitivity analysis was used to correlate density and clinical parameters (e.g., peri-implantitis stage). The assessment of systematic error showed no significant differences (*p* > 0.05). Expert consensus: ICC two-way random effects model.

## 3. Results

The results of assessing the bone tissue density of areas without pathological changes are shown in Table A3.

To assess the bone tissue density of the alveolar part of the mandible, areas of molars and premolars without signs of pathological changes in bone tissue were included. The number of comparison areas with the reference area (the angle of the lower jaw) was 55. The number of pixels in the selected comparison areas differed by no more than 10%. The average coefficient of variation of bone density for 55 measurements was 0.06 ± 0.01. The average coefficient of variation of the density estimate from 12 jaw angle measurements was 0.05 ± 0.01.

It has been shown that the assessment of the ratio of the average density of areas without signs of pathological changes in bone tissue to the average density calculated from the area selected in the area of the angle of the lower jaw has a lower coefficient of variation than separately selected areas in the lower jaw, which makes the choice of the angle of the jaw as reference is more preferable. In this case, the actual manual selection of the region is simplified, and it becomes possible to make a more correct comparison of relative densities, for example, during dynamic observation, due to the constancy of the coefficient of variation of the reference region.

The analysis showed that with manual selection of the reference and comparison areas for areas without signs of pathological changes in bone tissue, the average values of relative density ratios were 1.052, standard deviation 0.058, and coefficient of variation 0.055 ± 0.011%.

To assess the relative density of bone tissue in areas with various types of pathological changes, nine radiographs were examined. The distribution of patient radiographs is presented in Table A4, the results of the evaluation of indicators are in Table A5.

The compared areas were similar in statistical characteristics: standard deviations were 5–7% of the average value. The average relative bone density of the assessed defective areas to the jaw angle was 0.64 ± 0.11 (Table A5), and the average relative density of the areas without pathology to the jaw angle was 1.052 ± 0.058 (Table A3).

The indicator allows one to reliably assess changes in bone, and if there are differences in values of less than 11%, one can conclude that bone density has been completely restored.

### Clinical Case Study

An example of the practical use of relative bone density measurements to assess the healing process is presented.

ROIs were selected as recommended, statistical characteristics were calculated, and mean gray values were used to calculate relative bone density.

**Figure 4:**
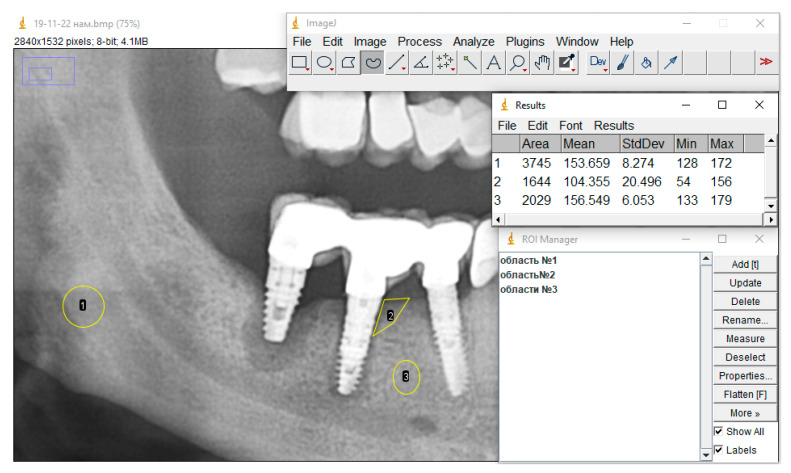
X-ray picture before treatment: area 1—reference, area 2—peri-implantitis, area 3—area without signs of pathological changes in bone tissue.

Figure 4 shows the X-ray picture before treatment of peri-implantitis, where area 1 is the reference, area 2 is peri-implantitis, and area 3 is the area without signs of pathological changes in the bone tissue.

The ratio of the bone density of a healthy bone area (without pathological changes) to the bone tissue density of the reference area is 1.019, and the ratio of the bone tissue density in the peri-implantitis zone to the density of the bone tissue of the reference area is 0.68.

**Figure 5:**
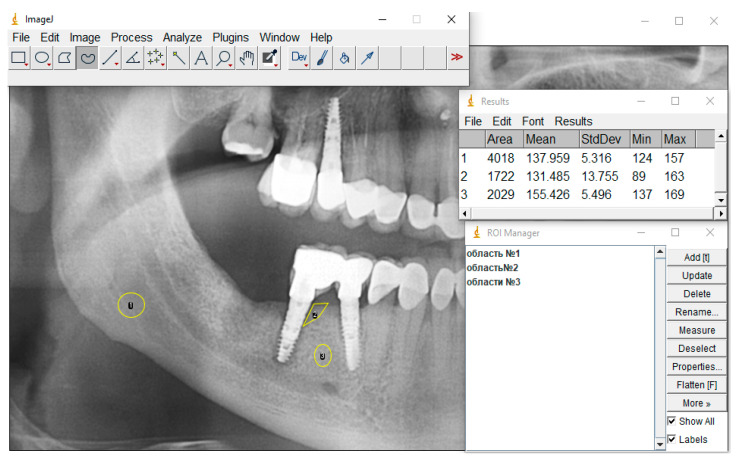
X-ray picture after treatment: where area 1 is the standard, area 2 is peri-implantitis, and area 3 is the area without signs of pathological changes in bone tissue.

Figure 5 shows the X-ray picture of the same patient 6 months after treatment for peri-implantitis, where area 1 is the standard, area 2 is peri-implantitis, and area 3 is the area without signs of pathological changes in bone tissue.

The values of the ratio of relative bone density in the peri-implantitis area to the reference area over 6 months changed from 0.68 to 0.95.

## 4. Discussion

Modern methods of digital processing of X-ray images allow the use of qualitative and quantitative measures of information, which makes it possible to detect changes in tissues, including bone tissue. Domestic and foreign literature offers protocols for assessing bone mass density using software products.

Ihan Hren N. and Miljavec M. (2008) used surrounding bone as a reference when measuring relative bone density. Compared to other sources [19,20], this method has advantages. However, such a selection of reference areas for estimating the relative density has a fairly high coefficient of variation of the average gray measurements and is a labor-intensive procedure. We conducted a study of the statistical characteristics of various areas without pathologies and found that the most preferable choice was the angle of the mandible as a reference area. It has been shown that the assessment of the ratio of the average density of areas without signs of pathological changes in bone tissue to the average density calculated from the area selected on the lower angle of the jaw has a lower coefficient of variation than separately selected areas in the lower jaw, which makes the choice of the angle of the jaw as a reference preferable. In this case, the actual manual selection of the region is simplified, and it becomes possible to make a more correct comparison of relative densities, for example, during dynamic observation, due to the constancy of the coefficient of variation of the reference region.

Determining the relative density threshold for deciding bone loss and recovery is not only statistically challenging, but also requires expert knowledge. The relative density value should tend to unity in the process of bone tissue restoration. As bone loss progresses, relative density decreases.

In domestic and foreign literature, decision-making criteria are not proposed; comparison of relative density values is carried out without taking into account the sensitivity of the indicator [4,7,9,10,12,17].

The research results showed that the average relative density of areas without pathology to our chosen standard is 1.052 ± 0.058, relative density values in the range of 94–106% of the standard (difference < 11%) correspond to clinical criteria for bone tissue restoration (*p* < 0.01, chi-square test).

The given clinical example clearly demonstrates the capabilities of the proposed assessment method. With a clear measurement protocol and correct choice of reference area, the obtained quantitative estimates of relative density make it possible to give a more accurate picture of bone regeneration, carry out dynamic monitoring, and predict treatment outcomes.

If there are sufficient sample sizes for specific types of pathology, with the use of experts, it is possible to determine thresholds by which the severity of bone loss can be classified.

Although manual ROI selection does introduce operator-dependent variability, our data show that with a strict protocol:

1. ICC > 0.9 for key regions;
2. CV < 5% for reference areas.

This is consistent with radiographic examination standards.

## 5. Conclusions

The proposed protocol using the ImageJ software package establishes a method for quantifying jaw bone density measurements. The method demonstrates good reproducibility indicators (ICC > 0.9) when the developed protocol is followed, which corresponds to modern standards of X-ray research. The proposed protocol provides a standardized approach to bone density assessment with reproducibility comparable to current alternative methods, but requires strict adherence to all processing steps.

## 6. Limitations

Although the main analysis showed sufficient power, the sample size for rare pathologies (e.g., stage III peri-implantitis) remains limited. For these subgroups, we used:

- A Bayesian approach with informed prior distributions;
- Sensitivity analysis with effect variation.

The main limitations of reproducibility were as follows:

1. Dependence on the quality of the original images;
2. Need for training on working with ROI (min. 10 training cases);
3. Influence of shooting parameters (requires calibration of the device).

## Appendix A

**Table A1:** Distribution of study areas.

Number of Areas	Location of the Study Area
9	3.5–3.4
10	3.6–3.5
10	3.7–3.6
8	4.5–4.4
9	4.6–4.5
9	4.7–4.6

**Table A2:** Distribution of panoramic radiographs with various types of changes in bone tissue.

No.	State of the Study Area
1	Condition before treatment of peri-implantitis
2	Observation after treatment of peri-implantitis
3	Condition before treatment of peri-implantitis
4	Observation after treatment of peri-implantitis
5	Condition before treatment of peri-implantitis
6	Observation after treatment of peri-implantitis
7	Condition before cystectomy
8	Condition before cystectomy
9	Control radiograph 6 months after surgery
10	Control radiograph 6 months after surgery
11	Condition before tooth extraction with periapical lesion
12	Condition before tooth extraction with periapical lesion
13	Condition before tooth extraction with periapical lesion
14	Condition before tooth extraction with periapical lesion
15	Control radiograph 6 months after extraction
16	Control radiograph 6 months after extraction
17	Control radiograph 6 months after extraction
18	Control radiograph 6 months after extraction

**Table A3:** Results of analysis of bone tissue areas without pathological changes.

Number of Areas	Location of the Study Area	Ratio of Averages Density of the Area of Interest/Density of the Jaw Angle	Standard Deviation	The Coefficient of Variation
9	3.5–3.4	1.082	0.048	0.044
10	3.6–3.5	1.068	0.064	0.059
10	3.7–3.6	1.008	0.059	0.058
8	4.5–4.4	1.087	0.053	0.048
9	4.6–4.5	1.063	0.035	0.032
9	4.7–4.6	1.011	0.033	0.032
Average value		1.052	0.058	0.055 ± 0.011

**Table A4:** Distribution of panoramic radiographs with various types of pathological changes.

No.	State of the Study Area
1	Condition before treatment of peri-implantitis
2	Condition before treatment of peri-implantitis
3	Condition before treatment of peri-implantitis
4	Condition before cystectomy
5	Condition before cystectomy
6	Condition before tooth extraction with periapical lesion
7	Condition before tooth extraction with periapical lesion
8	Condition before tooth extraction with periapical lesion
9	Condition before tooth extraction with periapical lesion

**Table A5:** Results of assessing parameters in patients with pathological changes in bone tissue.

No.	$S_{Reference}$	$m_{Reference}$	${std}_{Reference}$	$S_{Defect}$	$m_{Defect}$	${std}_{Defect}$	m2/m1
1	9928.00	178.00	8.65	9606.00	120.00	14.45	0.67
2	6144.00	194.40	13.86	5500.00	110.13	13.10	0.57
3	9155.00	197.00	11.00	1889.00	105.00	12.00	0.53
4	8900.00	142.00	7.90	2925.00	112.00	9.00	0.79
5	6564.00	143.70	8.00	2365.00	84.70	6.60	0.59
6	12,415.00	173.00	9.00	15,765.00	103.00	19.00	0.60
7	10,567.00	159.00	17.00	6889.00	115.00	13.00	0.72
8	3956.00	123.00	5.70	4013.00	98.00	8.50	0.80
9	5994.00	145.00	9.00	4420.00	68.00	19.00	0.47
Average values	8180.33	161.68	10.01	5930.22	101.76	12.74	0.64
Average coefficient							0.11

## References

[1] Gupta A., Devi P., Srivastava R., Jyoti B. (2014). Intra oral periapical radiography—Basics yet intrigue: A review. Bangladesh J. Dent. Res. Educ..

[2] Yau H.-T., Lin Y., Tsou L., Lee C. (2013). An Adaptive Region Growing Method to Segment Inferior Alveolar Nerve Canal from 3D Medical Images for Dental Implant Surgery. Comput.-Aided Des. Appl..

[3] Kumar R., Khambete N., Priya E. (2011). Extraoral periapical radiography: An alternative approach to intraoral periapical radiography. Imaging Sci. Dent..

[4] Chibisova M.A., Batyukov N.M. (2020). Methods of X-ray examination and modern radiation diagnostics used in dentistry. Inst. Dent..

[5] Moiseev D.A., Kopetsky I.S., Nikolskaya I.A., Ilyukhin G.S., Gazarov S.Y., Madatyan G.K., Sevastyanova V.V., Kurbatina A.B. (2023). The problem of primary infection in endo-periodontal lesions: A systematic review. Endod. Today.

[6] Moiseev D.A., Heigetyan A.V., Karammaeva M.R., Zadorozhny A.V., Zadorozhny M.A., Feoktistova D.V. (2024). A new method of intraperiopocket galvanophoresis as part of the complex therapy of fast-progressive periodontitis. Clin. Dent..

[7] Arzhantsev A.P. (2019). Radiological manifestations of jaw cysts. The Russian electronic. J. Radiat. Diagn..

[8] Natto Z.S., Almeganni N., Alnakeeb E., Bukhari Z., Jan R., Iacono V.J. (2019). Peri-Implantitis and Peri-Implant Mucositis Case Definitions in Dental Research: A Systematic Assessment. J. Oral Implantol..

[9] Shatta A., Bissada N.F., Ricchetti P., Paes A., Demko C. (2019). Impact of Implant and Site Characteristics on the Pattern of Bone Loss in Peri-implantitis. Int. J. Oral Maxillofac. Implant..

[10] Ryumshin R.A., Davydova O.B., Savvidi K.G., Piekalnits I.Y., Kostin I.O. (2020). Assessment of X-ray bone density in middle-aged patients with moderate chronic periodontitis. Tver Med. J..

[11] Doube M., Kłosowski M.M., Arganda-Carreras I., Cordelières F.P., Dougherty R.P., Jackson J.S., Schmid B., Hutchinson J.R., Shefelbine S.J. (2010). BoneJ: Free and extensible bone image analysis in ImageJ. Bone.

[12] Khojastepour L., Mohammadzadeh S., Jazayeri M., Omidi M. (2017). In vitro Evaluation of the Relationship between Gray Scales in Digital Intraoral Radiographs and Hounsfield Units in CT Scans. J. Biomed. Phys. Eng..

[13] Lemos A.D., Katz C.R., Heimer M.V., Rosenblatt A. (2014). Mandibular asymmetry: A proposal of radiographic analysis with public domain software. Dent. Press J. Orthod..

[14] Preus H.R., Torgersen G.R., Koldsland O.C., Hansen B.F., Aass A.M., Larheim T.A., Sandvik L. (2015). A new digital tool for radiographic bone level measurements in longitudinal studies. BMC Oral Health.

[15] Elsayed M.A. (2022). Multiparameter Image Analysis to Evaluate Dentinal Tubules Patency after Using Different Irrigation Protocols. Sci. Dent. J..

[16] NurFadhilah A., Masriadi, Mohammad D.U., Sarafin A., Mila F., Harun A., Sitti Fadhillah O.M., Amanah P., Karnila (2021). Comparision Analysis between of Software Imagej and Adaptive Region Growing Approach Android-Based in Determining the Width of Periapical Abscess Lesions. Ann. Rom. Soc. Cell Biol..

[17] Hisham S., Abdullah N., Mohamad N., Mohamad H., Franklin D. (2019). Quantification of secondary dentin formation using dental orthopantomographs in a contemporary Malaysian population. Aust. J. Forensic Sci..

[18] Williams C., Wu Y., Bowers D.F. (2015). ImageJ analysis of dentin tubule distribution in human teeth. Tissue Cell.

[19] Ihan H.N., Miljavec M. (2008). Spontaneous bone healing of the large bone defects in the mandible. Int. J. Oral Maxillofac. Surg..

[20] Geiger M., Blem G., Ludwig A. (2016). Evaluation of ImageJ for Relative Bone Density Measurement and Clinical Application. J. Oral Health Craniofac Sci..

